# Supplementary material for: Lipophilized Epigallocatechin Gallate Derivative Exerts Anti-Proliferation Efficacy through Induction of Cell Cycle Arrest and Apoptosis on DU145 Human Prostate Cancer Cells
Source: Nutrients. 2019 Dec 28;12(1):92. doi: 10.3390/nu12010092 (PMC7020104; doi:10.3390/nu12010092)
Supplement: Supplementary file 1 [file nutrients-12-00092-s001.pdf]

Supplementary Table 1. ^1H and ^{13}C Chemical Shifts (δ) of EGCG and Tetralauroyl EGCG

C/H position	EGCG		Tetralauroyl EGCG	
	^1H	^{13}C	^1H	^{13}C
2	5.11	78.55	5.20	77.60
3	5.51	70.00	5.49	69.55
4	2.89	25.42	2.96	26.64
	2.93		3.00	
5		157.49		157.39
6	6.06	96.59	6.06	96.89
7		157.99		157.90
8	6.01	95.69	6.01	95.93
9		156.40		156.89
10		99.27		99.28
1′		130.89		131.47
2′	6.56	106.58	7.00	106.94
3′		146.43		150.82
4′		133.68		131.50
5′		146.43		150.82
6′	6.56	106.58	7.00	106.94
1″		121.87		121.25
2″	7.05	108.78	7.45	108.90
3″		146.26		151.35
4″		139.19		137.87
5″		146.26		151.35
6″	7.05	108.78	7.45	108.90
COO		165.56		165.82

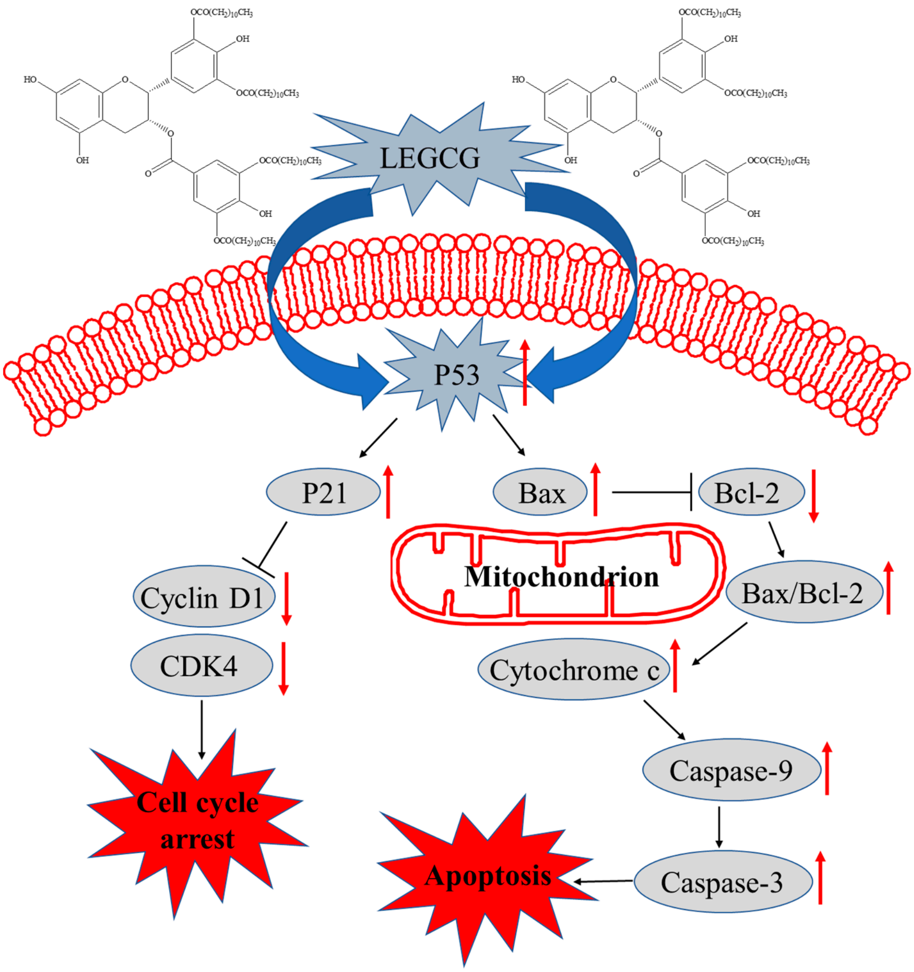

Supplementary Figure 1. The proposed signaling pathway triggered by LEGCG on DU145 cells.
